# Development and Initial Implementation of a Clinical Monitoring Strategy in a Non-regulated Trial: a research note from the ReStOre II Trial

**DOI:** 10.12688/hrbopenres.13763.3

**Published:** 2024-10-18

**Authors:** Linda O'Neill, Fiona Murphy, Derval Reidy, Camille Poisson, Juliette Hussey, Emer Guinan

**Affiliations:** 1 School of Medicine, Discipline of Physiotherapy, Trinity College, Dublin, Ireland; 2 Trinity St James's Cancer Institute, Trinity College, St James's Hospital, Dublin, Ireland; 3 School of Pharmacy and Pharmaceutical Sciences, Trinity College, Dublin, Ireland; 4 Wellcome-Health Research Board Clinical Research Facility, Trinity College, St James’s Hospital, Dublin, Ireland; 5 Research Innovation, Trinity College, Trinity College Dublin, the University of Dublin, Dublin, Ireland; 6 Trinity Innovation & Enterprise, Trinity College, Hartford, Connecticut, USA

**Keywords:** Monitoring, Non-regulated trial, Independent Data Monitoring Committee

## Abstract

**Background:**

Data and Safety Monitoring is integral to quality assurance of clinical trials. Although monitoring is a core legal component of regulated clinical trials, non-regulated trials are not mandated to incorporate monitoring. Consequently, the monitoring process has been underutilised and underreported in this setting. This research report outlines the development and plans for implementing a bespoke Clinical Monitoring Strategy within the ‘ *Rehabilitation Strategies Following Oesophagogastric and Hepatopancreaticobiliary Cancer (ReStOre II) Trial’*, a non-regulated trial comparing a 12-week multidisciplinary programme of rehabilitation to standard care in a cohort of 120 cancer survivors.

**Methods:**

This research note provides a detailed overview of the ReStOre II Clinical Monitoring Strategy and describes the development of the strategy pre and post awarding of the grant. The strategy consists of the establishment and implementation of a comprehensive trial governance structure, inclusive of a Trial Management Group, Trial Steering Committee Meeting, and Independent Data Monitoring Committee. In addition, external trial monitoring by the Clinical Research Facility at St James’s Hospital. Three monitoring visits will be conducted during the trial; i) site initiation visit, ii) interim monitoring visit, and iii) close our visit.

**Results:**

The Clinical Monitoring Strategy has been finalised and is currently being implemented within the ReStOre II Trial. Two site initiation visits and one interim monitoring visit have been completed to date.

**Conclusion:**

This research note provides a template for implementation of a Clinical Monitoring Strategy in a non-regulated clinical trial.

**Registration:**

ReStOre II Trial: https://clinicaltrials.gov/ct2/show/NCT03958019

## Introduction

Monitoring is a core strategy to ensure best practice, participant safety, integrity, internal validity and overall quality assurance of clinical trials ^1– 3^, and is a key aspect of the International Conference of Harmonisation Good Clinical Practice (ICH GCP), which provides a standard for the conduct of clinical trials ^4^. In Ireland, regulated clinical trials falls under the remit of the Health Product Regulation Authority (HPRA), and typically involve an Investigational Medicinal Product (IMP), regulated under the IMP regulatory framework (SI 190/2004), and therefore are legally required to comply with ICH GCP and the EU Clinical Trial Regulation EU#536/2014 or the EU Medical Device Regulation 2017/745 ^5^. Non-regulated trials are not legally required to comply with these regulations. The Declaration of Helsinki is a statement of ethical principles for medical research involving human subjects which is applicable to both regulated and non-regulated research, and for clinical trials is transposed into law through ICH GCP.

Trial monitoring involves independent review of trial documentation to ensure data is accurate and complete and verifiable against source documentation ^6^, procedures are compliant with the approved protocol, and that the trial is being conducted in line with ICH GCP ^4^. Trial monitoring can involve independent data monitoring committees (IDMC), independent statistical analysis, centralised monitoring, site monitoring, and endpoint adjudication ^2, 3^. External data monitoring may be responsive to metrics set at the outset of the trial ^7^, or routine. Responsive data monitoring may be just as effective as routine monitoring with fewer financial implications ^8^, although certain vulnerable populations, (e.g. paediatrics) may benefit from external data monitoring as standard, with cost merely a secondary consideration ^9^. Others argue that external data monitoring should not be done routinely. Instead, investigators should pay attention to the core principles of trial monitoring, and design rigorous monitoring procedures based on risk appropriate for each trial ^10^. External data monitoring is essential for phased clinical trials evaluating pharmacological agents wherein safety evaluation is a core feature ^11^. Since the Food & Drug Administration and the European Medicines Agency released guidelines in the 2000s ^12, 13^, a handful of studies have demonstrated efficacy for external data monitoring. Indeed, one review attributed benefits in trial recruitment rates and protocol compliance to monitoring ^3^.

The practicalities and efficacy of implementing monitoring strategies in non-regulated clinical trials is poorly documented. It stands to reason that the quality of trials involving complex interventions (e.g., behavioural, psychological, nursing, nutritional, exercise and rehabilitation), where ICH-GCP is not a legal requirement, may benefit from standardised monitoring procedures. The Rehabilitation Strategies Following Oesophagogastric and Hepatopancreaticobiliary Cancer (ReStOre II) trial is a two-armed randomised controlled trial (RCT), funded by a Health Research Board (HRB) Definitive Intervention and Feasibility Award (DIFA). The trial builds on findings from a HRB Health Research Award (2014–2017) which characterised unmet physical, nutritional and psychosocial needs in upper gastrointestinal cancer survivors ^14– 18^, leading to the development, feasibility testing and piloting of a tailored 12-week multidisciplinary rehabilitation programme ^19– 22^. The ReStOre II definitive RCT will examine if the 12-week programme can improve functional capacity and health related quality-of-life in 120 survivors of oesophagogastric and hepatopancreaticobiliary cancer in comparison to usual care ^23^. Recruitment occurs at St James’s Hospital and St Vincent’s University Hospital, Dublin, with all trial assessments and intervention visits completed at the St James’s Hospital Clinical Research Facility (SJH-CRF). The trial was due to begin recruitment early 2020 but this was delayed significantly due to COVID-19. The trial opened in Spring 2022 and is due for completion in Quarter 4 (Q4) 2024.

### Aim of research report

As a non-regulated trial, a novel aspect of ReStOre II is the inclusion of a bespoke Clinical Monitoring Strategy. The aim of this research report is to outline the development and initial implementation of the ReStOre II Clinical Monitoring Strategy. 

## Methods

### Pre-award planning for a clinical monitoring strategy

The ReStOre II Trial is funded through a HRB DIFA Award (HRB-DIFA-2018-009). The grant application included a Clinical Monitoring Strategy as a commitment to quality assurance.

The proposed Clinical Monitoring Strategy included:

1) A Trial Governance Structure:
  1. Trial Management Group
  2. Trial Steering Committee
  3. IDMC
2) A Trial Monitoring Plan
  1. Site initiation visit
  2. Interim monitoring visit
  3. Close out visit

The monitoring proposal was developed in consultation with the Quality and Regulatory Affairs (QRA) Department in the SJH-CRF. As ReStOre II would not be investigating an IMP or any medical devices beyond the remit of their marketing authorisation, it was classified as a non-regulated trial which did not require an application to the HPRA. Accordingly, it was anticipated that three monitoring visits (Site Initiation Visit, Interim Monitoring Visit, and Close Out Visit) would likely be sufficient to ensure quality control, estimated to require 15 days of monitoring. This proposal was included in the infrastructural agreement with the SJH-CRF as part of the grant application and appropriate budget allocated.

### Post award development of the clinical monitoring strategy

The HRB-DIFA award was awarded to one of the authors (JH) in 2018. Upon commencement of the award on 1 ^st^ May 2019, the details of the Clinical Monitoring Strategy were developed and consistent with the pre-award proposal, this included a Trial Governance Structure and a Trial Monitoring Plan.

***Developing trial governance structures.*** A governance structure comprising three trial management committees for project management and oversight were established. The first version of the Terms of Reference (TOR) for each committee was finalised on the 29 ^th^ August 2019.

The Trial Management Group TOR outlined that the committee would meet monthly for the first six months of the trial, and quarterly thereafter, with additional meetings scheduled as required. Membership of the ReStOre II Trial Management Group consists of the Principal Investigator (PI), Co-applicants x3, Project Manager, Trial Statistician, and the Research Team (research assistants, research nurses). The Trial Management Group has responsibility for the day-to-day running of the trial, initial development of the study protocol, the review and approval of subsequent protocol amendments and the submission of reports to the Trial Steering Committee.

The Trial Steering Committee TOR outlined that the committee would provide independent oversight and supervision to the trial and ensure that the trial is conducted in accordance with Research Governance guidelines. The ReStOre II Trial Steering Committee would consists of n=4 Independent Members, n=1 patient representative, the PI, n=2 Co-applicants, and the Project Manager. The Trial Steering Committee would be chaired by an Independent Member and meets biannually. The committee has responsibility for the formal approval of the trial protocol, monitoring of trial progress against anticipated timelines and targets, and monitoring of adherence to the trial protocol and patient safety through consideration of reports from the IDMC. 

Finally, the TOR for the IDMC outlined that the committee would consist of a panel of experienced researchers, who are independent of the trial, with complementary expertise in clinical exercise oncology (n=1), medical statistics (n=1), dietetics (n=1), upper gastrointestinal cancer surgery (n=1) and quality and regulatory affairs (n=1). The IDMC would meet biannually and have collective responsibility for monitoring the progress of the trial from the start to the end of data collection, scrutinising recruitment, randomisation, retention, protocol compliance and adherence and assessing adverse events. The IDMC would review the monitoring reports produced by the SJH-CRF following planned visits outlined in the Trial Monitoring Plan and may make recommendations to the Trial Steering Committee based on their review of the monitoring reports.

***Developing the trial monitoring plan.*** The ReStOre II research team worked with the SJH-CRF QRA Department to develop the Trial Monitoring Plan. This commenced with an overall study risk assessment and a second risk assessment specifically to evaluate the level of monitoring required.

An overall study risk assessment was completed according to standard QRA Department procedures and deemed RESTORE II to be a low-risk trial. Subsequently, the QRA Department used the SCTO Risk Based Monitoring Score Calculator ^24^. The SCTO calculator determines the monitoring strategy for a clinical trial in consideration of the level of risk across twenty-three risk factors organised into seven categories. The SCTO risk-based monitoring score calculator classified ReStOre II as a low-risk trial (see *Extended Data* ^25^) and accordingly the QRA Department confirmed that the three monitoring visits, originally envisaged at pre-award stage (site initiation visit, interim monitoring visit, and close out visit) would be adequate. A Trial Monitoring Plan version 1 (2020) and version 2 (2023 ( *see Extended Data*)) were established by the QRA Department in line with this low-risk status.

All monitoring visits were planned to be conducted by a member of the SJH-CRF QRA Department who is independent of the ReStOre II trial team but performs sponsor delegated tasks from Trinity College Dublin.

The Trial Monitoring Plan incorporated two site initiation visits, one at St James’s Hospital and one at St Vincent’s University Hospital. The site initiation visit was planned to occur prior to ‘greenlighting’ the trial for screening and recruitment at both sites to ensure compliance with the following:

1. All necessary approvals e.g., ethics, hospital approval are in place
2. All planned trial procedures are GCP compliant and compliant with the approved protocol
3. All trial staff are trained appropriately, and training documentation is stored appropriately
4. Adequate and appropriate resources are in place for the trial
5. Insurance cover is in place for the trial
6. The investigator site file is set up and managed appropriately
7. All trial documentation is ready for trial commencement including the trial protocol, case report forms, deviation log, incident report forms, participant identification log, screening documentation, data management plan, and randomisation plan.
8. Trial Management, Governance and Safety TOR is in place.

The Trial Monitoring Plan includes an interim monitoring visit at St James’s Hospital, the intervention site, to ensure continued quality control during the intervention, continued adherence to GCP during the intervention, and continued adherence to the approved protocol during the intervention. Accordingly, the Trial Monitoring Plan schedules the interim monitoring visit early in the trial, and as soon as possible after the first cohort of patients are enrolled onto the trial. The interim monitoring visit includes review of the following documents for existence and accuracy: eligibility screening and enrolment logs, informed consent forms (existence, version control, signatures and dates), the consent process, source data, randomisation records, investigator site file maintenance, protocol amendments and approvals, protocol deviation log, incident log and incident reporting, staff training records, equipment calibration and service records, and confidential patient identification log. Source data verification of data within the case report form and other data collection systems is conducted on the first participant, and up to 20% of the cohort enrolled at the time of the interim monitoring visit. This involves reviewing the paper case report forms against the source data (e.g., study visit notes), raising data queries and flagging missing or inaccurate data captured. The interim monitoring visit concludes with a meeting between the monitor and the study team to discuss findings. Additional interim monitoring visits can be completed if required.

Following completion of the ReStOre II trial, an in-person close out visit will be conducted by the monitoring team in the SJH-CRF on request. The close-out visit will verify the existence of original informed consent forms for enrolled subjects, collection of all Case Report Forms, and collection and completion of safety data. Any outstanding issues from prior monitoring visits will be resolved and the monitoring team will remind the site study team of their responsibilities for record retention and inspect the investigator site file for completeness prior to study closure.

## Results

### Implementation of the clinical monitoring strategy

The Clinical Monitoring Strategy for the ReStOre II Trial has achieved the following deliverables to date (June 2023):

Trial Governance Deliverables

1. Establishment of the Trial Management, Governance and Safety Monitoring TOR (29 ^th^ August 2019).
2. Implementation of the Trial Management, Governance and Safety Monitoring TOR. Between award commencement (May 2019) and June 2023 the following number of management group meetings have occurred: Trial management Group (n=18), Trial Steering Committee (n=5), IDMC (n=3).

Trial Monitoring Plan Deliverables

1. Finalisation of the Trial Monitoring Plan (18 ^th^ January 2022).
2. The site initiation visit was conducted in St James’s Hospital on the 18 ^th^ January 2022, with greenlight received on the 3 ^rd^ February 2022.
3. The site initiation visit was conducted in St Vincent’s University Hospital on the 11 ^th^ January 2023, with greenlight received on the 20 ^th^ January 2023.
4. The interim monitoring visit was conducted on the 7 ^th^ February 2023 at St James’s Hospital and included the following: review of investigator site file, source document/ case report form review for n=7 participants, all informed consent forms were reviewed for completeness and accuracy, a review of enrolment status, review of protocol violations, incident forms, and intervention accountability. A number of minor action points were identified and addressed by the site team in writing on the 23 ^rd^ February 2023 and the monitoring team in the SJH-CRF confirmed on the 24 ^th^ February 2023 that they were satisfied with the site teams’ response to all action points.
5. Version 2 of the Trial Monitoring Plan by the SJH-CRF QRA Department on the 26 ^th^ June 2023.

The following deliverables are ongoing:

1. The Trial Management Group will continue to meet quarterly, and the Trial Steering Committee and IDMC biannually for the remainder of the trial (due for completion Q4 2024).
2. Following completion of the trial a close out visit will be conducted by the SJH-CRF monitoring team on request.

## Discussion

This research note outlines the development and initial implementation of the Clinical Monitoring Strategy for the ReStOre II Trial (a non-regulated trial) in collaboration with a SJH-CRF. We will produce a final report describing complete implementation of this strategy upon closure of the trial (Q4 2024). Monitoring provides a quality framework and ensures that the principles of the Declaration of Helsinki and relevant ICH GCP principles are followed. Monitoring helps assure patient safety and data integrity for non-IMP/Medical Device trials. It also trains researchers to consider quality at each step of the research process and trains them to work on studies where ICH-GCP is mandatory (IMP/Medical Device trials). All research staff involved in the ReStOre II trial complete ICH GCP training as a mandatory training requirement. Through working with the SJH-CRF and QRA team, the research team understands the importance of Good Documentation, version control, and updating of the Participant Information Leaflet and Informed Consent Form documentation for ethics applications. Increasing the awareness and utilisation of monitoring strategies within non-regulated trials may be useful for improving the quality and validity of non-regulated trials going forward.

## Data Availability

All data underlying the results are available as part of the article and no additional source data are required. Open Science Framework: Development and initial Implementation of a Clinical Monitoring Strategy in a Non-regulated Trial: a research note from the ReStOre II Trial https://doi.org/10.17605/OSF.IO/NMX3G ^25^ This project contains the following extended data: ReStOre II Study Risk Assessment Form signed.pdf ReStOre II Trial Monitoring Plan version 2. pdf Data are available under the terms of the Creative Commons Zero "No rights reserved" data waiver (CC0 1.0 Public domain dedication).
