# Supplementary material for: Development and Initial Implementation of a Clinical Monitoring Strategy in a Non-regulated Trial: a research note from the ReStOre II Trial
Source: HRB Open Res. 2024 Oct 18;6:46. Originally published 2023 Sep 8. [Version 3] doi: 10.12688/hrbopenres.13763.3 (PMC11401978; doi:10.12688/hrbopenres.13763.3)
Supplement: Peer review history

# Reviewer report: Reviewer response for version 3

**DOI:** 10.21956/hrbopenres.15336.r42833

**Published:** 2024-10-23

**Authors:** Ahmad Ozair

**Affiliations:** 1 Department of Neurosurgery, University of Maryland Medical System, Baltimore, Maryland, USA

I thank the authors for constructively engaging in peer-review. The manuscript is now suitable.

Is the work clearly and accurately presented and does it cite the current literature?

Partly

If applicable, is the statistical analysis and its interpretation appropriate?

Not applicable

Are all the source data underlying the results available to ensure full reproducibility?

No source data required

Is the study design appropriate and is the work technically sound?

Yes

Are the conclusions drawn adequately supported by the results?

No

Are sufficient details of methods and analysis provided to allow replication by others?

No

Reviewer Expertise:

I am able to assess the entirety of the article. I have a formal medical background with experience and education in clinical trials, clinical research, oncology, and biostatistics.

I confirm that I have read this submission and believe that I have an appropriate level of expertise to confirm that it is of an acceptable scientific standard.

# Reviewer report: Reviewer response for version 2

**DOI:** 10.21956/hrbopenres.15166.r37350

**Published:** 2024-09-14

**Authors:** Sharon B. Love

**Affiliations:** 1 MRC Clinical Trials Unit at UCL, Institute of Clinical Trials and Methodology, University College London, London, England, UK

Thank you to the authors for updating the manuscript. I have no further comments.

Is the work clearly and accurately presented and does it cite the current literature?

Partly

If applicable, is the statistical analysis and its interpretation appropriate?

Not applicable

Are all the source data underlying the results available to ensure full reproducibility?

No source data required

Is the study design appropriate and is the work technically sound?

Yes

Are the conclusions drawn adequately supported by the results?

Yes

Are sufficient details of methods and analysis provided to allow replication by others?

Yes

Reviewer Expertise:

I am a researcher in clinical trial monitoring I lead the UKCRC Monitoring group and the TMRP Monitoring group. I have been the trial statistician on many trials

I confirm that I have read this submission and believe that I have an appropriate level of expertise to confirm that it is of an acceptable scientific standard.

# Reviewer report: Reviewer response for version 2

**DOI:** 10.21956/hrbopenres.15166.r37349

**Published:** 2024-09-14

**Authors:** Mandy Jackson, Pooja Varghese

**Affiliations:** 1 RCSI Sponsorship Office, Royal College of Surgeons in Ireland, Dublin, Ireland

happy that previous comments have been addressed

Is the work clearly and accurately presented and does it cite the current literature?

Partly

If applicable, is the statistical analysis and its interpretation appropriate?

Not applicable

Are all the source data underlying the results available to ensure full reproducibility?

Yes

Is the study design appropriate and is the work technically sound?

Yes

Are the conclusions drawn adequately supported by the results?

Yes

Are sufficient details of methods and analysis provided to allow replication by others?

Partly

Reviewer Expertise:

Reviewer able to assess entirety of the article. Background in clinical monitoring, quality, pharmacovigilance, regulatory affairs and nursing.

We confirm that we have read this submission and believe that we have an appropriate level of expertise to confirm that it is of an acceptable scientific standard.

# Reviewer report: Reviewer response for version 2

**DOI:** 10.21956/hrbopenres.15166.r41350

**Published:** 2024-08-01

**Authors:** Ahmad Ozair

**Affiliations:** 1 Department of Neurosurgery, University of Maryland Medical System, Baltimore, Maryland, USA

This review pertains to the revised version (version 2) of a paper describing the monitoring of a non-regulated trial called ReStOre II conducted in Ireland, where non-regulated refers to trials that are not required to comply with ICH GCP and EU Regulations, in accordance with the IMP regulatory framework (analogous to the FDA IND/IDE process). The monitoring described in this research note is related to the risk aspects of the trial.

 Given below are my comments in no particular order.

 1. First, given the limited word count of 2500 words, authors should heavily cut short the introduction and focus on describing methods and results. More use of EXTENDED DATA should be done.

 2. I share the concerns by Prof. Sharon Love that were provided for version 1. The focus of the manuscript is the clinical monitoring strategy, which I don't believe is described in sufficient detail that external individuals (within and outside Ireland) can sufficiently learn from.

 3. Prof. Love previously recommended the use of subheadings for structuring the methods and results. While the authors have carried out minor revisions to their manuscript in response, they need to formally use subheadings to reduce word count while adding greater detail.

 4. " committee would consist monthly " - the wrong usage needs correction.

 5. Where abbreviation is used once only, please remove the abbreviation to avoid unnecessary jargon.

 6. Please consider providing the last available protocol, patient information leaflet, and consent form in the extended data. If not possible, at least the protocol should be provided. 

 7. Some sentences are unsupported by the evidence presented. Their 'conclusion' like style needs to be overhauled or toned down.

 e.g. "Our experience of developing and implementing this strategy has greatly enhanced the quality and rigor of this non-regulated trial."

 I don't think this N-of-1, uncontrolled intervention provides sufficient proof that this strategy alone "greatly enhanced the quality and rigor". 

 8. Vague, unnecessary, or redundant statements should be removed to make space for a greater description of the methods and results.

 e.g."Although there is no legal requirement for these studies to be monitored, monitoring is an extremely important part of the research cycle, particularly within a teaching hospital setting. "

 This is already described in the introduction. 

 9. If the authors have space, consider adding some discussion of FDA guidance and regulations in this regard.

Is the work clearly and accurately presented and does it cite the current literature?

Partly

If applicable, is the statistical analysis and its interpretation appropriate?

Not applicable

Are all the source data underlying the results available to ensure full reproducibility?

No source data required

Is the study design appropriate and is the work technically sound?

Yes

Are the conclusions drawn adequately supported by the results?

No

Are sufficient details of methods and analysis provided to allow replication by others?

No

Reviewer Expertise:

I am able to assess the entirety of the article. I have a formal medical background with experience and education in clinical trials, clinical research, oncology, and biostatistics.

I confirm that I have read this submission and believe that I have an appropriate level of expertise to confirm that it is of an acceptable scientific standard, however I have significant reservations, as outlined above.

# Reviewer report: Reviewer response for version 1

**DOI:** 10.21956/hrbopenres.15055.r36711

**Published:** 2023-11-01

**Authors:** Sharon B. Love

**Affiliations:** 1 MRC Clinical Trials Unit at UCL, Institute of Clinical Trials and Methodology, University College London, London, England, UK

This is a paper describing the setup of the monitoring for a non-regulated trial called ReStOre II. It includes the set up of three committees as well as on-site monitoring. The monitoring is related to the risk aspects of the trial.

 As the authors say, it is useful to know how others have set up their monitoring and the basis of the decisions that were made. There is still not much literature about the practicalities of monitoring and even less in non-regulated trials. This paper is to be applauded.

 My main concern is with the methods section of the paper. The methods section to me jumps around. It would be far more useful and encourage readers to continue reading if it was in a logical order. I would like to know that a trial monitoring plan was envisaged, grant funding was obtained and how it was implemented. Whereas in this paper, this text

*The Trial Monitoring Plan for the ReStOre II trial consists of the following monitoring strategies:*

*1. Site initiation visit*

*2. Interim monitoring visit*

*3. Close out visit*

*4. Trial management group meetings (Trial Management Group and Trial Steering Committee)*

*5. IDMC*

 Occurs in the middle of the methods. It would be more useful at the beginning. This is in a different order and using different terminology than when it was introduced in the methods. Whichever order is chosen, it is easier to read if the order remains the same throughout the document and the methods section is written in that same order.  Maybe part of the results paragraph is about how the clinical monitoring strategy changed from grant applications to how you did it in practice.

**Minor points**

 Ref 4 – please could you give an url

 Prof Hussey – replace by author initials, “one of the authors (JH)

 Establishment of the Trial Management, Governance and Safety Monitoring Terms of Reference – the paragraph on IDMC does not detail that the committee members are independent of the trial – I think its worth repeating although the word independent is in the title.

 Results – PIL – needs defining

 Results – ICF – needs defining

 Acknowledgements – I guess you also meant to thank members of the IDMC

Is the work clearly and accurately presented and does it cite the current literature?

Partly

If applicable, is the statistical analysis and its interpretation appropriate?

Not applicable

Are all the source data underlying the results available to ensure full reproducibility?

No source data required

Is the study design appropriate and is the work technically sound?

Yes

Are the conclusions drawn adequately supported by the results?

Yes

Are sufficient details of methods and analysis provided to allow replication by others?

Yes

Reviewer Expertise:

I am a researcher in clinical trial monitoring I lead the UKCRC Monitoring group and the TMRP Monitoring group. I have been the trial statistician on many trials

I confirm that I have read this submission and believe that I have an appropriate level of expertise to confirm that it is of an acceptable scientific standard, however I have significant reservations, as outlined above.

# Response

**Published:** 2023-12-01

**Authors:** Emer Guinan

**Affiliations:** Department of Surgery, Trinity College, Dublin, Dublin, Ireland

This is a paper describing the setup of the monitoring for a non-regulated trial called ReStOre II. It includes the set up of three committees as well as on-site monitoring. The monitoring is related to the risk aspects of the trial.

 As the authors say, it is useful to know how others have set up their monitoring and the basis of the decisions that were made. There is still not much literature about the practicalities of monitoring and even less in non-regulated trials. This paper is to be applauded.

**Thank you for your complimentary feedback on the value of this paper.**

 My main concern is with the methods section of the paper. The methods section to me jumps around. It would be far more useful and encourage readers to continue reading if it was in a logical order. I would like to know that a trial monitoring plan was envisaged, grant funding was obtained and how it was implemented. Whereas in this paper, this text

 The Trial Monitoring Plan for the ReStOre II trial consists of the following monitoring strategies:

 1. Site initiation visit

 2. Interim monitoring visit

 3. Close out visit

 4. Trial management group meetings (Trial Management Group and Trial Steering Committee)

 5. IDMC

 Occurs in the middle of the methods. It would be more useful at the beginning. This is in a different order and using different terminology than when it was introduced in the methods. Whichever order is chosen, it is easier to read if the order remains the same throughout the document and the methods section is written in that same order.  Maybe part of the results paragraph is about how the clinical monitoring strategy changed from grant applications to how you did it in practice.

**Thank you for this helpful feedback. We have tried to apply your feedback and restructure the methods to improve readability and flow. We hope that these edits improve the paper.**

 Minor points

 Ref 4 – please could you give an url

**A URL has been provided for reference 4 which has also been updated to the latest version of these guidance.**

 Prof Hussey – replace by author initials, “one of the authors (JH)

**Prof Hussey has been replaced with ‘One of the authors (JH)’**

 Establishment of the Trial Management, Governance and Safety Monitoring Terms of Reference – the paragraph on IDMC does not detail that the committee members are independent of the trial – I think its worth repeating although the word independent is in the title.

**Thank you for this point – the word independent has been added as appropriate to that paragraph.**

 Results – PIL – needs defining

 Results – ICF – needs defining

**Definitions of PIL and ICF have both been included.**

 Acknowledgements – I guess you also meant to thank members of the IDMC

**Apologies for omitting to list the members of the IDMC. Their names have now been included in the acknowledgements section.**

**A number of other minor edits have been made to the manuscript to keep within word count limits.**

# Reviewer report: Reviewer response for version 1

**DOI:** 10.21956/hrbopenres.15055.r36117

**Published:** 2023-09-29

**Authors:** Mandy Jackson, Pooja Varghese

**Affiliations:** 1 RCSI Sponsorship Office, Royal College of Surgeons in Ireland, Dublin, Ireland

This article was very interesting and is a very under researched area. Most academic Sponsors do not have the resources to conduct monitoring in unregulated studies so it was interesting to read about the impact such monitoring had on the Restore II trial.

 For the above question: "Are sufficient details of methods and analysis provided to allow replication by others?"-this was answered as partly because at this point, the reviewer is of the opinion that further research in the area is needed (as implied in the discussion).

 Please see below some constructive feedback divided into the sections as they appear in the article:

 INTRODUCTION

- Reference is made to the International Conference on harmonisation. This has been changed to the International Council on harmonisation and GCP guideline that was current at the time of the article was Revision 2. I believe reference #4 therefore should be updated.
- In the introduction, the following sentence does not read correctly:
  “In Ireland, regulated clinical trials falls under the remit of the Health Product Regulation Authority (HPRA), and typically involve an Investigational Medicinal Product (IMP) or Medical Device, regulated under the IMP regulatory framework (SI 190/2004),”
  SI 190 2004 is specifically governing clinical trials involving IMP and not medical devices so please consider revising this sentence
- In the introduction, second paragraph reference to ICH GCP (#3) does not correspond with the reference provided in the reference section. Should this reference be #4?
- In Introduction para 2, “Trial monitoring can involve independent data monitoring committees (IDMC), independent statistical analysis, centralised monitoring, site monitoring, and endpoint adjudication”. Sentence not referenced so reviewer unable to verify if this sentence is factual.
- Introduction para 3, “The practicalities and efficacy of implementing monitoring strategies in non-regulated clinical trials is poorly documented. It stands to reason that the quality of trials involving complex interventions (e.g., behavioural, psychological, nursing, nutritional, exercise and rehabilitation), where ICH-GCP is not a legal requirement, may benefit from standardised monitoring procedures”. Sentence not referenced so reviewer unable to verify if this sentence is factual.

METHODS

- If the close out visit will verify the existence of original informed consent forms for enrolled subjects, this may be difficult to achieve remotely (as proposed). Would the team consider an in person close out visit in order to achieve the objectives outlined in the last paragraph of the methods section?
- In the Finalising the Trial Monitoring Plan section in the methods section it reads “Any outstanding issues from prior monitoring visits will be resolved and the monitoring team will remind the site study team of their responsibilities for record retention and inspect the investigator site file for completeness prior to study closure” As per GCP record retention is the responsibility of the sponsor and the Investigator and not solely the site staff. This responsibility can be delegated by the investigator to site staff so please consider adding wording to clarify this in the sentence.

DISCUSSION

- “The aim of this research report is to outline the development and initial implementation of the ReStOre II Clinical Monitoring Strategy” the discussion implies there is more to the aim than just to outline the development and initial implementation of the strategy but that the authors were also investigating the impact of the monitoring strategy on the research team’s awareness of good clinical practice and the quality & rigor of the data. Should the aims be changed?

Is the work clearly and accurately presented and does it cite the current literature?

Partly

If applicable, is the statistical analysis and its interpretation appropriate?

Not applicable

Are all the source data underlying the results available to ensure full reproducibility?

Yes

Is the study design appropriate and is the work technically sound?

Yes

Are the conclusions drawn adequately supported by the results?

Yes

Are sufficient details of methods and analysis provided to allow replication by others?

Partly

Reviewer Expertise:

Reviewer able to assess entirety of the article. Background in clinical monitoring, quality, pharmacovigilance, regulatory affairs and nursing.

We confirm that we have read this submission and believe that we have an appropriate level of expertise to confirm that it is of an acceptable scientific standard, however we have significant reservations, as outlined above.

# Response

**Published:** 2023-12-01

**Authors:** Emer Guinan

**Affiliations:** Department of Surgery, Trinity College, Dublin, Dublin, Ireland

This article was very interesting and is a very under researched area. Most academic Sponsors do not have the resources to conduct monitoring in unregulated studies so it was interesting to read about the impact such monitoring had on the Restore II trial.

 For the above question: "Are sufficient details of methods and analysis provided to allow replication by others?"-this was answered as partly because at this point, the reviewer is of the opinion that further research in the area is needed (as implied in the discussion).

 Please see below some constructive feedback divided into the sections as they appear in the article:

**Thank you for your positive summary of the value of this article. Your constructive feedback is greatly appreciated.**

 INTRODUCTION

 Reference is made to the International Conference on harmonisation. This has been changed to the International Council on harmonisation and GCP guideline that was current at the time of the article was Revision 2. I believe reference #4 therefore should be updated.

**Thank you for highlighting this updated reference. Reference #4 has been updated to International Council for Harmonisation of Technical Requirements for Pharmaceuticals for Human Use (ICH). ICH Harmonised Guideline: Integrated Addendum to ICH E6(R1): Guideline for Good Clinical Practice E6(R2). December 1, 2016. Available from: https://www.ema.europa.eu/documents/scientific-guideline/ich-guideline-good-clinical-practice-e6r2-step-5_en.pdf**

 In the introduction, the following sentence does not read correctly:

 “In Ireland, regulated clinical trials falls under the remit of the Health Product Regulation Authority (HPRA), and typically involve an Investigational Medicinal Product (IMP) or Medical Device, regulated under the IMP regulatory framework (SI 190/2004),”

 SI 190 2004 is specifically governing clinical trials involving IMP and not medical devices so please consider revising this sentence

**Thank you for highlighting this point – the words ‘or medical device’ has been removed from this sentence.**

 In the introduction, second paragraph reference to ICH GCP (#3) does not correspond with the reference provided in the reference section. Should this reference be #4?

**Thank you for highlighting this error – reference #3 in the second paragraph has been updated to reference #4.**

 In Introduction para 2, “Trial monitoring can involve independent data monitoring committees (IDMC), independent statistical analysis, centralised monitoring, site monitoring, and endpoint adjudication”. Sentence not referenced so reviewer unable to verify if this sentence is factual.

**References #2 and #3 have been added as references to support this statement.**

 Introduction para 3, “The practicalities and efficacy of implementing monitoring strategies in non-regulated clinical trials is poorly documented. It stands to reason that the quality of trials involving complex interventions (e.g., behavioural, psychological, nursing, nutritional, exercise and rehabilitation), where ICH-GCP is not a legal requirement, may benefit from standardised monitoring procedures”. Sentence not referenced so reviewer unable to verify if this sentence is factual.

**We include this sentence as a summary of the evidence presented in the introduction and state it as our own conclusion which gives rise to the need for this paper. We do not feel that it requires a specific reference.**

 METHODS

 If the close out visit will verify the existence of original informed consent forms for enrolled subjects, this may be difficult to achieve remotely (as proposed). Would the team consider an in person close out visit in order to achieve the objectives outlined in the last paragraph of the methods section?

**Thank you for this suggestion and following consultation with our team we have agreed that we can include an in-person close out visit in our plan.**

 In the Finalising the Trial Monitoring Plan section in the methods section it reads “Any outstanding issues from prior monitoring visits will be resolved and the monitoring team will remind the site study team of their responsibilities for record retention and inspect the investigator site file for completeness prior to study closure” As per GCP record retention is the responsibility of the sponsor and the Investigator and not solely the site staff. This responsibility can be delegated by the investigator to site staff so please consider adding wording to clarify this in the sentence.

**Thank you for raising this point. This is an academic trial and therefore it is Investigator lead study and some sponsor tasks are delegated to the sponsor. The TMF is combined with the ISF and the responsibility of maintaining the ISF/TMF falls under the investigator.**

 DISCUSSION

 “The aim of this research report is to outline the development and initial implementation of the ReStOre II Clinical Monitoring Strategy” the discussion implies there is more to the aim than just to outline the development and initial implementation of the strategy but that the authors were also investigating the impact of the monitoring strategy on the research team’s awareness of good clinical practice and the quality & rigor of the data. Should the aims be changed?

**This is an interesting point and in hindsight it would have been useful to have added in measures of impact but no, at present, we have no plans to measure the impact of the monitoring strategy on the research team. We state in the discussion that all members of the research team complete mandatory training in ICH-GCP but we do not measure any outcomes from that training.**
